# Estradiol Regulates mRNA Levels of Estrogen Receptor Beta 4 and Beta 5 Isoforms and Modulates Human Granulosa Cell Apoptosis

**DOI:** 10.3390/ijms22095046

**Published:** 2021-05-10

**Authors:** Alice Pierre, Anne Mayeur, Clémentine Marie, Victoria Cluzet, Jonathan Chauvin, Nelly Frydman, Michael Grynberg, Joelle Cohen-Tannoudji, Céline J. Guigon, Stéphanie Chauvin

**Affiliations:** 1 BFA, UMR 8251, CNRS, ERL U1133, Inserm, Université de Paris, F-75013 Paris, France; alice.pierre@inserm.fr (A.P.); marie.clementine.lp@orange.fr (C.M.); victoria.cluzet@gmail.com (V.C.); michael.grynberg@aphp.fr (M.G.); joelle.cohen-tannoudji@univ-paris-diderot.fr (J.C.-T.); celine.guigon@univ-paris-diderot.fr (C.J.G.); 2 Service de Médecine de la Reproduction et Préservation de la Fertilité, Hôpital Antoine Béclère, 92140 Clamart, France; anne.mayeur@aphp.fr (A.M.); nelly.frydman@aphp.fr (N.F.); 3 Lixoft, 8 rue de la Renaissance, Bâtiment D, 92160 Antony, France; jonathan.chauvin@lixoft.com

**Keywords:** estrogen receptor, isoforms, human granulosa cells, apoptosis

## Abstract

Estrogen receptor beta (ERβ) plays a critical role in granulosa cell (GC) functions. The existence of four human ERβ splice isoforms in the ovary suggests their differential implication in 17β-estradiol (E2) actions on GC apoptosis causing follicular atresia. In this study, we investigated whether E2 can regulate ERβ isoforms expression to fine tune its apoptotic activities in human GC. For this purpose, we measured by RT-qPCR the expression of ERβ isoforms in primary culture of human granulosa cells (hGCs) collected from patients undergoing in vitro fertilization, before and after E2 exposure. Besides, we assessed the potential role of ERβ isoforms on cell growth and apoptosis after their overexpression in a human GC line (HGrC1 cells). We confirmed that ERβ1, ERβ2, ERβ4, and ERβ5 isoform mRNAs were predominant over that of ERα in hGCs, and found that E2 selectively regulates mRNA levels of ERβ4 and ERβ5 isoforms in these cells. In addition, we demonstrated that overexpression of ERβ1 and ERβ4 in HGrC1 cells increased cell apoptosis by 225% while ERβ5 or ERβ2 had no effect. Altogether, our study revealed that E2 may influence GC fate by specifically regulating the relative abundance of ERβ isoforms mRNA to modulate the balance between pro-apoptotic and non-apoptotic ERβ isoforms.

## 1. Introduction

The vast majority of ovarian follicles is eliminated by the physiological process of follicular atresia. At the antral stage, atresia essentially results from the apoptosis of granulosa cells (GCs) [1,2]. During each menstrual cycle, a cohort of antral follicles is selected to further grow, but usually a single follicle achieves its growth and maturation up to ovulation. The acquisition of follicular dominance is not based on morphometric criteria but rather on the potential of this follicle to secrete high concentrations of estradiol (E2) upon sufficient stimulation by follicle-stimulating hormone (FSH), arising from the acquisition of a higher sensitivity of GC to FSH [3]. Consistently, atretic antral follicles exhibit lower levels of E2 in the follicular fluid as compared to dominant follicles [4]. Interestingly, studies on rhesus monkeys showed that capsules of E2 placed subcutaneously for 24 h to deliver a high amount of E2 in the circulation (~300 pg/mL) induce atresia of the original dominant follicle, independently of FSH deprivation [5]. In addition, these studies demonstrated that E2 reduced both GC viability and steroid secretion of these follicles, suggesting that E2 produced by the dominant follicles may regulate atresia of the other follicles [6]. Nonetheless, the real implication of E2 in follicle atresia remains ambiguous since contradictory findings were reported in other species, such as in rodents, wherein E2 would decrease follicular atresia [7,8].

**Figure 1:**
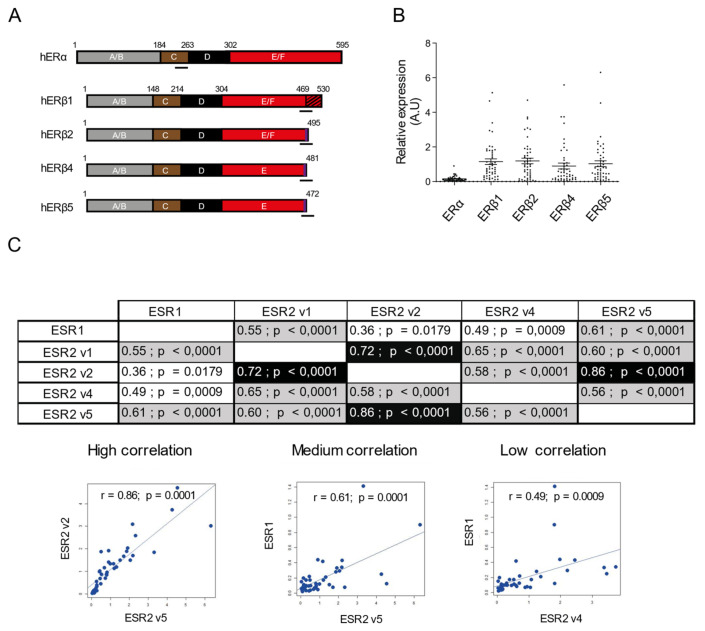
ERβ isoform mRNAs are highly expressed in hGCs with positive correlations between pairs of ERβ isoforms. (**A**) Schematic representation of ovarian human ERα and ERβ (hER). The A/B domain at the NH2 terminus contains the AF-1 site, where other transcription factors interact. The C/D domain (brown/black boxes) contains the two-zinc finger structure that binds to DNA, and the E/F domain (red box) contains the ligand binding pocket as well as the AF-2 domain, which directly contacts coactivator peptides. ERβ spliced isoforms are formed from alternative splicing of the last coding exon (shown by the striped bar); ERβ isoforms are identical in their first 468 amino acids but differ in the sequence corresponding to the end of the ligand binding of ERβ1. Approximate locations of the primers used for qPCR are indicated. (**B**) hGCs were cultured for 24 h before RNA extraction. Relative levels of ERα (*ESR1*) and ERβ1 (*ESR2* v1), ERβ2 (*ESR2* v2), ERβ4 (*ESR2* v4), and ERβ5 (*ESR2* v5) isoforms mRNAs were determined by RT-qPCR analysis for each patient (*n* = 49). Transcript levels were normalized to *GAPDH* transcripts abundance. Values are represented as means ± SEM from two or three identical wells per patient, measured in triplicate. (**C**) We evaluated the correlations between relative mRNAs levels of each pair of genes. Data are represented in the table; correlation values are categorized as “high” r > 0.65 (black highlighted), “medium” r between 0.5 and 0.65 (grey highlighted), and “low” r < 0.5. An example of the relationship between mRNA abundances for each category is represented by the fitted (log) blue line (----). Significance values from Pearson correlations are given on the graphs.

E2 actions are predominantly mediated by the steroid estrogen receptors ERα and ERβ, which are encoded by two distinct genes *ESR1* and *ESR2*, respectively producing mature transcripts containing eight exons. Both ERs belong to the large superfamily of nuclear receptors, which mostly function as ligand-dependent transcription factors. Within follicles, ERα is highly expressed in theca cells whereas ERβ is the predominant form in GCs [9]. ERβ then arises as the main mediator of E2 actions in GCs, although the cooperation of ERβ with ERα is required for maintaining the differentiation state of GCs [10]. The discovery of various ERβ isoforms further provides evidence for a higher degree of complexity in E2 signaling. To date, there are two ERβ isoforms in mice [11] and five isoforms (ERβ1–5) in humans (Figure 1A) generated through species-specific alternating splicing of exon 8 [12], therefore limiting the use of rodent models to study the roles of hERβ isoforms. In the human ovary, all ERβ isoforms except ERβ3 (testis-specific) are expressed, with ERβ4 being the less abundant one [13,14]. A single immunohistochemical study performed at an early stage of folliculogenesis described ERβ1, ERβ2, and ERβ4 in GCs of human primary follicles [12]. However, no information on ERβ isoform abundance in primary or antral follicles was included [12]. At the molecular level, the full-length ERβ1 form is able to bind ligands while this feature is absent in human C-terminal modified/truncated ERβ2, ERβ4, and ERβ5 isoforms [15]. Ligand-activated ERα and ERβ1 form homo- or heterodimers to regulate specific gene transcription by interacting directly with estrogen-response elements (EREs) usually located upstream from the promoter, or indirectly by tethering to other transcription factors, such as activating protein 1 (AP-1) or specificity protein (SP-1) enhancer elements [16]. This interaction rapidly regulates the transcription of estrogen-responsive genes. Because of the alteration of the 3-D structure of the C-terminal ligand binding domain, ERβ2, ERβ4, and ERβ5 are deficient in ligand-dependent transactivation activity, making ERβ1 the only functional receptor isoform [15]. Nonetheless, ERβ4 and ERβ5 can heterodimerize with ERβ1 under physiological circulating E2 concentrations and can exert dominant positive effects on E2-induced ERβ1 transactivation in HEK293 cells [15]. ERβ2, ERβ4, and ERβ5 were also described to form heterodimers with ERα to repress ligand-activated ERα-mediated transcriptional activity [17,18,19,20].

Therefore, in the current study, we sought to examine the nature of ERβ isoforms expressed in GC from preovulatory follicles collected from patients undergoing oocyte harvest for in vitro fertilization. We further investigated whether E2 can modulate the expression of its own receptors to fine tune its activities. We also analyzed ERβ isoform transactivities in a granulosa cell context and studied their potential role on cell viability using an immortalized human granulosa cell line (HGrC1).

## 2. Results

### 2.1. Positive Correlations between Pairs of ERβ Isoform mRNA Levels in hGCs

When we started our investigations, no quantitative data on the level of expression of each ERβ isoform in GCs from preovulatory follicles were available so far. Hence, we cultured freshly isolated and purified granulosa cells (hGCs) from preovulatory follicles collected during oocyte retrieval in patients undergoing in vitro fertilization. Forty-nine patients were recruited for this study. We measured the relative expression of *ESR1* (encoding ERα) and *ESR2* (encoding ERβ) isoforms (1, 2, 4, and 5) in hGCs by RT-qPCR, using *GAPDH* for normalization. PCR efficiency values of *ESR1*, *ESR2* (v1, v2, v4, and v5) and *GAPDH* were over 90% (see Table 1 in Section 4.8). We were unable to accurately quantify the expression of each ERβ isoform at the protein level (not shown) because of the absence of reliable specific antibodies so far. As shown in Figure 1B, we confirmed that ERβ1, ERβ2, ERβ4, and ERβ5 mRNA are predominantly present in cultured hCGs, when compared to ERα.

Next, we wanted to examine whether there could be a relationship between the levels of transcript of these receptors in hGCs. Correlation analyses between the five receptors mRNA abundance measured from these patients were performed using Monolix©Lixoft-Suite2020R1 software (Figure 1C). Strikingly, we found a systematic correlation between the relative mRNA levels of each pair of ERs (Figure 1C). Based on the pattern of correlation values, they were categorized into three groups: high (r > 0.65, in black), medium (0.5 > r > 0.65, in grey), or low correlation (r < 0.5) (Figure 1C). The results are summarized in the table shown in Figure 1C, which also exemplified pairwise scatterplots of the correlation between receptors’ mRNA levels corresponding to each of the three groups. We observed a high positive correlation between the relative mRNA levels of ERβ2 isoform with those of ERβ5 and ERβ1 isoforms (r = 0.86, *p* < 0.0001 and r = 0.72, *p* < 0.001, respectively). ERβ1 isoform mRNA levels also presented a medium positive relationship with those of ERβ4 (r = 0.65, *p* = 0.0001) and ERβ5 (r = 0.60, *p* = 0.0001) isoforms (Figure 1C). Besides, ERβ4 isoform mRNA levels positively correlated with those of ERβ5 isoform (r = 0.56, *p* < 0.0001). Regarding ERα mRNA levels, the relative transcript abundance of ERα was positively correlated with those of ERβ5 (r = 0.61, *p* = 0.0001) and ERβ1 (r = 0.55, *p* = 0.0001) isoforms. We did not observe other significant (r > 0.5) correlations between mRNA levels of ERα and those encoding ERβ isoforms (Figure 1C).

Overall, our analyses pointed towards the existence of various positive correlations between the mRNA levels of the different ERβ isoforms.

### 2.2. E2 Promotes Estrogen Receptor Transactivations in hGCs

**Figure 2:**
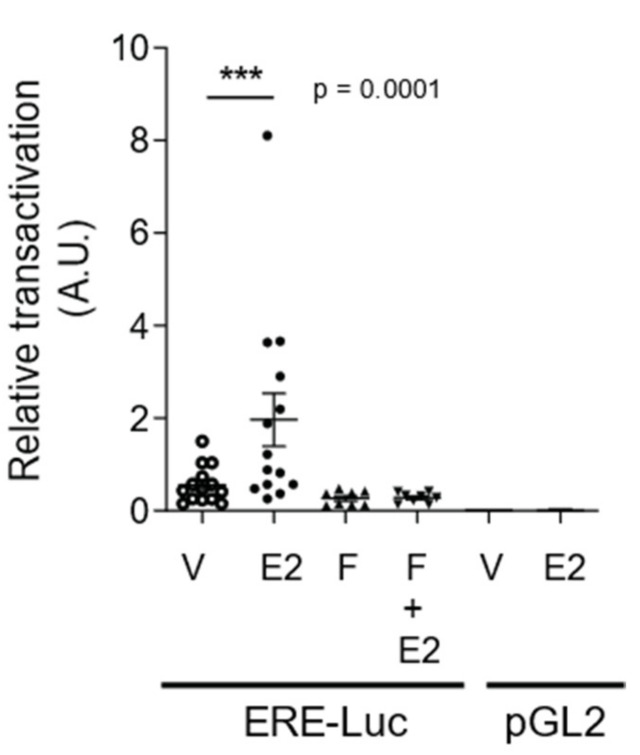
E2 induces transactivation of ERs in hGCs. Cells were transiently transfected with Firefly luciferase reporter plasmid containing or not (pGL2) estrogen-response elements (EREs) upstream of luciferase (ERE-Luc). An internal control vector containing Renilla luciferase was also included. Twenty-four hours post-transfection, cells were treated with either solvent vehicle (V) or 10 nM E2, in the presence or absence of 1 µM Fulvestrant (F), an ER degrader (SERD). Twenty-four hours later, cells were lysed and analyzed for luciferase activity. Firefly luciferase activity was normalized relative to Renilla luciferase activity, reported as relative transactivation activities arbitrarily set at 1 for the solvent vehicle (V) condition in ERE-Luc-transfected cells. Each point represents the mean ± SEM of 14 experiments (from each patient) performed in six replicates. *** *p* ≤ 0.001 vs. solvent vehicle (V) by two sides Wilcoxon signed-ranks test. A.U., Arbitrary Units.

To further assess whether E2 can promote biological activities in hGCs, we first ascertained that endogenous ERs were able to transactivate an estrogen reporter construct in these cells. For this purpose, cultured hGCs from follicles of 14 patients were transfected with an ERE-Luc or a control (pGL2) vector to measure their luciferase activity with 10 nM E2 treatment during 24 h. We chose this concentration to reproduce the intrafollicular concentration measured in follicular fluids of antral follicles [21,22]. In hGCs transfected with the ERE-Luc, pairwise comparison of vehicle (V, solvent) and E2-treated hGCs from each patient showed that E2 stimulated by 3.2-fold (*p* < 0.001) the ERE-dependent luciferase activity (Figure 2). E2 did not have any effect on hGCs transfected with the promoter-less pGL2 vector. Interestingly, the selective ER down regulator (SERD) Fulvestrant (F) prevented E2-induced luciferase activity, suggesting that this effect is ER dependent. Treatment with F alone did not significantly affect the basal luciferase activity. Our data demonstrate that E2 can activate ER signaling in cultured hGCs to elicit gene transactivation.

### 2.3. E2 Selectively Increased mRNA Levels of ERβ4 and ERβ5 Isoforms in hGCs

**Figure 3:**
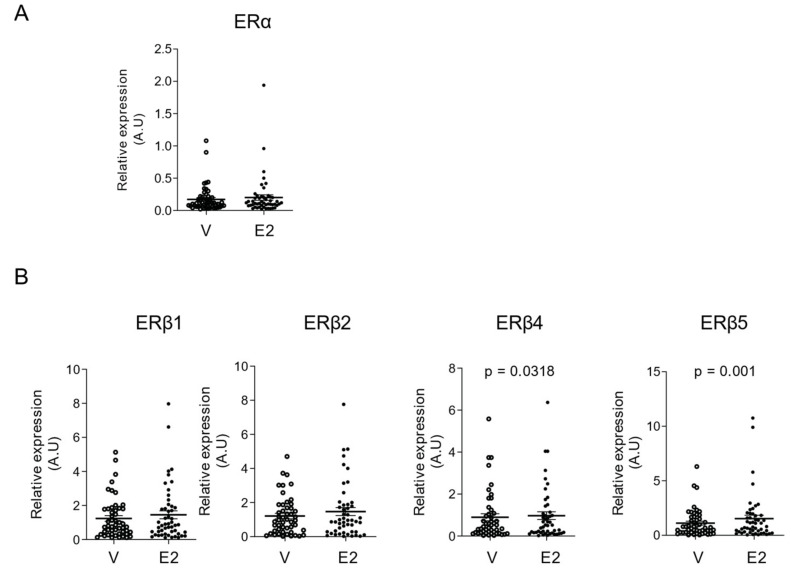
E2 selectively stimulates ERβ4 and ERβ5 isoforms’ mRNA abundance in hGCs. Cells were treated with solvent vehicle (V) or 10 nM E2 for 24 h. mRNAs were extracted and mRNA levels of ERα (**A**) and ERβ (1, 2, 4, and 5) isoforms (**B**) were measured relative to that of GAPDH by RT-qPCR. E2 significantly stimulates the relative transcript levels of ERβ4 and ERβ5 isoforms (*n* = 49). Values are represented as means ± SEM from two or three identical wells per patient, measured in triplicate. Statistical analysis was performed by the two-side Wilcoxon signed ranks test. A.U., Arbitrary Units.

Since hGCs express various ERβ isoform transcripts, we next wondered whether E2 could selectively regulate the mRNA abundance of ERβ isoforms. hGCs were cultured in steroid-depleted FCS growing medium for 24 h and treated with E2 or vehicle (V, solvent) for another 24 h. The mRNA levels of ERα as well as those of ERβ isoforms were measured by RT-qPCR, and normalized to those of GAPDH (Figure 3). Pairwise comparison of vehicle and E2-treated hGCs from each patient revealed that E2 significantly increased the mRNA level of ERβ4 isoform (*p* = 0.0318), and stimulated more significantly that of ERβ5 isoform (*p* = 0.001) when compared to the vehicle treatment (Figure 3B). E2 did not modify the abundance of ERα or ERβ1 and ERβ2 isoform mRNA (Figure 3A,B). These data clearly demonstrate that E2 selectively increases the mRNA level of ERβ4 and ERβ5 isoform in hGCs. Hence, by upregulating specific ERβ isoforms’ mRNA abundance in hGCs, E2 may act in an autocrine/paracrine manner to modulate its biological activities.

### 2.4. Absence of Influence of ERβ2, ERβ4, or ERβ5 on E2-Induced ERβ1 Transactivation in HGrC1-Transfected Cells

**Figure 4:**
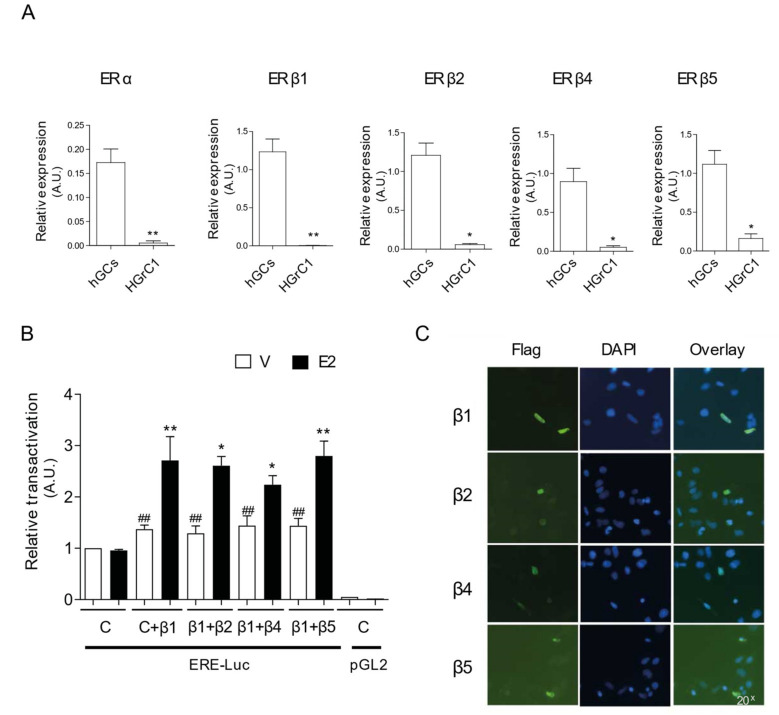
Absence of influence of ERβ2/β4/β5 on E2-induced ERβ1 transactivation in HGrC1-transfected cells. (**A**) The presence of ERα and ERβ (1, 2, 4, and 5) isoforms mRNA in hGCs (*n* = 49) and HGrC1 cells (*n* = 6) was evaluated by RT-qPCR (expression normalized to GAPDH). Values are represented as means ± SEM from three identical wells per patient, measured in triplicate. * *p* ≤ 0.05; ** *p* ≤ 0.01 by the Mann–Whitney test. A.U., Arbitrary Units. HGrC1 cells express low levels of endogenous ERα and ERβ isoforms, when compared to those expressed in hGCs. (**B**) HGrC1 cells were transiently cotransfected with the control Flag vector (C) alone or with the Flag-ERβ1 vector (β1), together with the Firefly luciferase reporter plasmid containing or not (pGL2) estrogen-response elements (EREs) upstream of luciferase (ERE-Luc). Flag-ERβ1 was also cotransfected (1:1 ratio) with either Flag-ERβ2 (β1+β2), Flag-ERβ4 (β1+β4), or Flag-ERβ5 (β1+β5). Renilla luciferase reporter plasmid was included as a normalizing transfection control. Twenty-four hours after solvent vehicle (V) or 10 nM E2 treatment, cells were lysed and analyzed for luciferase activity. Firefly luciferase activity was normalized to that of Renilla luciferase and reported as relative transactivation activities arbitrarily set at 1 for the control (C) solvent vehicle (V) condition in ERE-Luc-transfected cells. Each point represents the mean ± SEM in six replicates performed four times. * *p* ≤ 0.05; ** *p* ≤ 0.001 vs. vehicle (V) for each group of cotransfection, and ## *p* ≤ 0.001 vs. control (C) vehicle (V) using the Mann–Whitney test. A.U., Arbitrary Units. (**C**) HGrC1 cells were transiently transfected with either Flag-ERβ1 (β1), Flag-ERβ2 (β2), Flag-ERβ4 (β4), or Flag-ERβ5 (β5). Twenty-four hours post-transfection, cells were fixed, permeabilized, and the localization of Flag-ERβ proteins was monitored by immunofluorescence using an anti-Flag antibody (green). Nuclei were stained with DAPI (blue). Representative images at 20x magnification are presented.

Then, we wanted to decipher the effect of ERβ2, ERβ4, and ERβ5 isoforms on the transcriptional activity of the fully functional ERβ1 isoform in GCs. For this, we used an immortalized human granulosa cells line, the HGrC1 cells, which possess hallmarks of GCs (e.g., anti-Müllerian hormone (AMH), aromatase and Forkhead box protein L2 (FOXL2) expression) [23]. However, we observed that these cells displayed very weak levels of ERα and ERβ isoform mRNAs when compared to hGCs (from ~25- to 7-fold lower), with no detection (near detection limits) of ERβ1 mRNA (Figure 4A). These minute levels of endogenous ER mRNAs could explain the absence of E2-induced ER-mediated luciferase (ERE-Luc) activity that we observed in ERE-Luc-transfected HGrC1 cells (C) (Figure 4B). Therefore, we sought to assess whether overexpression of ERβ1 in HGrC1 cells would mediate transcriptional activation of the ERE-luciferase gene after E2 treatment, and whether cotransfection of ERβ2, ERβ4, or ERβ5 with ERβ1 (1:1 ratio) would regulate E2-induced ERβ1 transactivation in these cells. For this purpose, we constructed Flag-tagged ERβ (β1, β2, β4, and β5) fusion proteins to overcome the absence of specific antibodies for all ERβ isoforms. As expected [24], immunofluorescence performed on liposome-transfected HGrC1 exhibited tagged ERβ isoforms in the nucleus, as shown by the co-staining with DAPI (Figure 4C).

HGrC1 cells acquired E2 responsiveness when we overexpressed Flag-ERβ1 (β1) (Figure 4B). Indeed, Figure 4B showed a significant induction in luciferase activity upon E2 treatment (by ~2-fold, *p* = 0.008) in Flag-ERβ1-transfected cells. However, cotransfection of Flag-ERβ1 with the ligand-insensitive Flag-ERβ2, Flag-ERβ4, or Flag-ERβ5 with a 1:1 ratio did not significantly modify Flag-ERβ1 transactivity after E2 treatment (Figure 4B). We also observed a significant (*p* = 0.008) estrogen-independent transactivation of Flag-ERβ1 in vehicle-treated Flag-ERβ1-transfected cells, when compared to that of control (C, Flag vector) transfected cells. This ligand-independent activity was maintained when Flag-ERβ1 was cotransfected with Flag-ERβ2, Flag-ERβ4, or Flag-ERβ5. The promoter-less pGL2 construct did not show luciferase activity with or without the presence of E2 (Figure 4B).

### 2.5. ERβ1, ERβ2, ERβ4, and ERβ5 Differentially Affect HGrC1 Apoptosis

**Figure 5:**
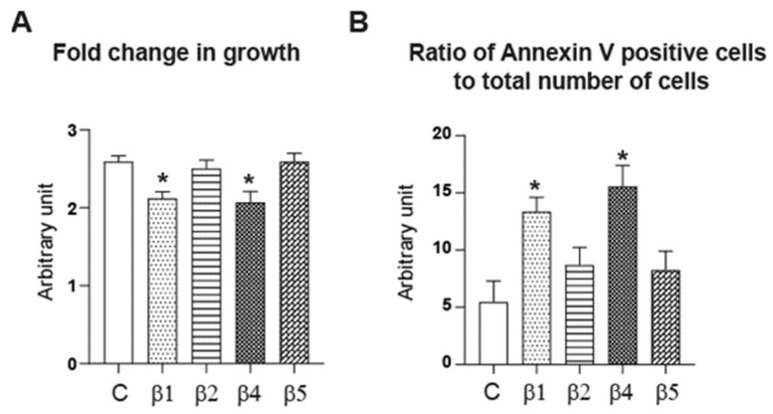
Contrary to ERβ5 and ERβ2, both ERβ1 and ERβ4 promote HGrC1 cell apoptosis. HGrC1 were transiently and efficiently (~70%) transfected by nucleofection with either Flag (C), Flag-ERβ1 (β1), Flag-ERβ2 (β2), Flag-ERβ4 (β4), or Flag-ERβ5 (β5). (**A**) MTT assay was performed 24 h and 48 h post-transfection. The quantity of formazan, which is directly proportional to the number of viable cells, was measured by recording changes in absorbance at 575 nm. Fold change in growth was expressed as the ratio of the OD_575nm_ value at 48 h post-transfection onto that measured at 24 h post-transfection. Contrary to Flag-ERβ5 (β5) or Flag-ERβ2 (β2), Flag-ERβ4 (β4) as well as Flag-ERβ1 (β1) significantly reduced the fold change in cell growth. Each experiment was performed 3-6 times, and data are reported as means ± SEM from four replicates. * *p* ≤ 0.05 vs. control Flag (C) by the Mann–Whitney test. (**B**) Twenty-four hours post-transfection, annexin V cell binding was assessed followed by measurement of the total cell number. Values represent the ratio between annexin V binding to the total cell number. Contrary to Flag-ERβ5 (β5) or Flag-ERβ2 (β2), Flag-ERβ4 (β4) as well as Flag-ERβ1 (β1) significantly increased cell apoptosis. Means ± SEM in four replicates performed 3–6 times * *p* ≤ 0.05 vs. control Flag (C) by the Mann–Whitney test.

Given that E2 stimulated the expression of both ERβ4 and ERβ5 in hGCs (Figure 3B), we wondered what could be the effect of overexpressing these two receptor isoforms on HGrC1 cell growth. To address this issue, we proceeded to HGrC1 cell nucleofection to achieve a high transfection rate (~70% efficiency), and started to study the biological effects of protein overexpression 24 h later, which corresponds to the recovery time after electroporation. HGrC1 cells were transfected with either Flag-ERβ4 (β4), Flag-ERβ5 (β5), or Flag C vector as a control. Besides, we used additional controls in these experiments by transfecting Flag-ERβ1 (β1) or Flag-ERβ2 (β2), whose expression was not modified by E2 treatment in hGCs (Figure 3B). We verified the efficiency of Flag-ERs transfection by immunofluorescence and RT-qPCR and observed a strong expression of each fusion protein (increase by 98.3 ± 2.8-fold, *p* < 0.001 for Flag-ERβ1; by 113 ± 5.4-fold, *p* < 0.05 for Flag-ERβ2; by 133 ± 6.7-fold, *p* < 0.05 for Flag-ERβ4; or by 131.5 ± 6.4-fold *p* < 0.05 for Flag-ERβ5, as compared with the control C condition). Interestingly, in HGrC1 cells transfected with Flag-ERβ5, we found a significant increase in endogenous ERβ2 mRNA expression (by 133.03 ± 7.18-fold as compared with control C, *p* < 0.05), supporting the potential functional interplay between these two ERβ isoforms in GCs. Then, we compared the number of viable HGrC1 cells present at 48 h post-transfection (before plasmid loss upon cell division) to that present at 24 h post-transfection, using tetrazolium salt assay (MTT) (Figure 5A).

As shown in Figure 5A, Flag-ERβ1 overexpression significantly reduced the growth of transfected HGrC1 cells, as revealed by the noticeable decrease (by ~18%, *p* = 0.02) in the fold change in growth when compared to control C, as expected [25]. Of note, these experiments were performed with media containing serum, indicating the contribution of growth factors and low levels of E2 [26] in these processes. Remarkably, overexpression of Flag-ERβ4 (β4) led to the same effect as that observed with Flag-ERβ1 (β1), with a marked reduction (by ~20%, *p* = 0.02) in the fold change in the growth of transfected HGrC1 cells. Conversely, HGrC1 cell growth was not affected by Flag-ERβ5 (β5) or Flag-ERβ2 (β2) overexpression, when compared to control C (Figure 5A).

The decrease in cell growth observed in HGrC1 cells transfected with Flag-ERβ1 and Flag-ERβ4 could result from increased apoptosis. To examine this possibility, we analyzed the staining of an early apoptotic marker, annexin V, in transfected HGrC1 cells with either Flag-ERβ1 (β1), Flag-ERβ2 (β2), Flag-ERβ4 (β4), Flag-ERβ5 (β5), or Flag vector (C) as control, and followed 24 h later with the binding of annexin V onto phosphatidylserines that are present on the outer leaflet of the cell membrane only during the first steps of cell apoptosis. Annexin V staining was normalized to the total viable cell number. Figure 5B shows that overexpression of Flag-ERβ1 (β1) as well as Flag-ERβ4 (β4) significantly increased cell apoptosis assessed by annexin V cell membrane binding (by ~224% and ~284%, respectively, *p* = 0.0286) whereas overexpression of Flag-ERβ5 (β5) or Flag-ERβ2 (β2) did not modify the cell membrane structure when compared to the control. Overall, our data demonstrate that overexpression of ERβ isoforms triggers different biological effects in HGrC1 cells, with ERβ1 and ERβ4 exhibiting pro-apoptotic activities while ERβ5 and ERβ2 do not play a role in this process.

## 3. Discussion

In our study, we used primary culture of purified luteinized GCs (hGCs), which remains the only human primary culture cell model of GCs so far. Importantly, we provided strong evidence that among the four ovarian ERβ isoforms, E2 selectively regulates the abundance of ERβ4 and ERβ5 isoform mRNA. In addition, we demonstrated that depending on the nature of the ERβ isoform, its overexpression in HGrC1 cells differently orientates cell fate, with ERβ1 and ERβ4 acting as pro-apoptotic factors. Our data thus suggest that E2 can modulate the relative mRNA abundance of ERβ isoforms in GCs, thereby possibly modulating ER protein levels to promote GC apoptosis, and consequently regulate follicle fate.

Our results demonstrated that all ovarian ERβ isoform mRNA levels are markedly higher than those of ERα in hGCs. We also revealed various positive correlations between the relative mRNA levels of each pair of ERs in hGCs. Especially, we observed a strong positive correlation between ERβ2 isoform mRNA levels with those encoding ERβ5 and ERβ1, and to a lesser extent a positive correlation between ERβ4 isoform mRNA levels with those of ERβ5. Accordingly, we detected an upregulation of ERβ2 mRNA levels following ERβ5 overexpression in HGrC1 cells, suggesting that these two isoforms may preferentially form heterodimers. In fact, ERβ isoforms were described to form heterodimers with each other in vitro [14]. Our findings also suggest the existence of common trans-acting factors or stimuli that control pairs of ERβ isoform mRNA abundance that might contribute to common physiological outcomes. These types of transcriptional regulations within receptor family members have already been described for the vasoactive peptide endothelin system, which contributes to many aspects of physiology and cell function, in particular controlling follicular rupture during ovulation [27]. In preovulatory follicles, endothelin-2 is the principal endothelin isoform expressed in human GCs together with its two distinct receptors (ETA and ETB), which possess opposite activities [28,29]. Both ETA and ETB expression is regulated by common stimuli, e.g., ovarian hormones (E2 and progesterone) [30] or inflammatory mediators [31], and their functional cooperation was reported to be essential in modulating endothelin physiological outcomes (vasoconstriction or vasodilatation) [27].

The precise molecular mechanism regulating ERβ alternative splicing is largely unknown. Recent data suggest that alternative hERβ promoter regulations may participate in these processes [32,33]. Indeed, different studies reported that the *ESR2* gene can be transcribed from three distinct untranslated first exons, termed exons 0K, 0N, and E1, which are spliced to the exon 1 [33,34,35]. Besides, the activity of these three promoters is finely regulated in a cell type-specific manner [32,34], with the 0K promoter being specifically activated by E2 in MCF-7 cells [33]. Smith and colleagues [32,33] showed that the translational efficiency of a GFP reporter gene was higher when the promoter contained the exon 0N sequence, also highlighting the importance of translational regulations in determining the expression of ERβ isoforms. In addition to these regulations, it is established that mRNA stability is controlled at its 3’-UTR through micro-RNAs (miR) binding. Regarding ER mRNA, miR-206 and miR-92 are reported to interact with the 3’-UTR regions of ERα and ERβ1 isoform mRNA, respectively, to trigger their degradation/cleavage [36]. In return, miR-206 and miR-92 stabilities are modulated by E2 in MCF-7 cells [36,37,38], thereby constituting an indirect pathway for E2 to regulate gene expression. In hGCs, we demonstrated that E2 selectively enhanced ERβ4 and ERβ5 isoform mRNA abundance, which could result from extensive and diverse regulations, such as direct or indirect effects of E2 on specific promoter activities and/or through downregulation of specific miRs. When we analyzed in silico the −3 kb 5’-flanking region of human *ESR2* promoter using the Jaspar database (considering a score >8), we detected five potential AP-1 sites (JUN/FOS) (at −2.585, −2.453, −2.437, −1.540, and −0.3 kb), two potential SP-1 sites (at −1.570 and −0.438 kb), and two potential ERE sequences (at −1.447 and −1.163 kb) upstream of the first exon. Since ERβ and ERα are able to interact directly or indirectly with these sequences, one could speculate that these various sites might be the target of ERs after E2 activation and could therefore participate in the transcriptional regulation of ERβ mRNA. Nonetheless, additional mechanisms involving tissue-specific proteins that regulate the recruitment of spliceosome components might further contribute to the induction of specific alternative splicing of ERβ isoforms.

ER functions are known to be interconnected with other hormone receptors that are expressed in GCs, such as androgen (AR), progesterone (PR), or thyroid hormone (TR) receptors. Crosstalk between this superfamily of nuclear receptors may contribute to E2 regulations in GCs. Indeed, E2 was described in the uterus to regulate AR expression, with AR amplifying E2 signaling [39]. In addition, PR isoforms expression were depicted to be dependent on E2 in the ovary [40]. Finally, since the consensus DNA sequences bound by ER and TR share a common half site, and TR can regulate gene transcription in the absence of ligand [41], it is possible that competition between the two receptors may antagonize the other’s effect [42].

Since ERβ1 possesses an intact ligand binding domain, it can trigger E2 effects through genomic mechanisms whereas ERβ2, ERβ4, and ERβ5, which are unable to bind ligands and have no innate activities of their own, might only influence gene transcription through their dimerization with ERβ1 [15]. As expected, we showed that ERβ1 displayed transactivity upon E2 treatment in HGrC1 cells. However, ERβ2, ERβ4, and ERβ5 did not alter E2-induced ERβ1 transactivity in HGrC1 cells, in contrast to what has been reported in HEK293 cells [15]. Therefore, the ability of ERβ isoforms to exert dominant positive effects on ERβ1 activity may be cell specific and may not be operative in GCs. In addition, in our experiments, we exposed HGrC1 cells to E2 at 10 nM to mimic ovarian follicles’ environment [22], which are concentrations 10 times higher than those found in the circulation and usually used in other studies [15]. Hence, one could speculate that depending on the E2 concentration, ERβ signaling might be different.

Furthermore, our results provided evidence that overexpressing ERβ4 in HGrC1 cells induced cell apoptosis, to the same extent as that observed with the well-described pro-apoptotic ERβ1 [25,43,44]. Contrary to ERβ1, which probably promotes its pro-apoptotic effects through ligand-dependent or ligand-independent genomic mechanisms in HGrC1 cells, ERβ4 might rather promote its effect via non-genomic mechanisms, for instance, through cytoplasmic signaling cascades, as already described for ERβ5 [45]. Indeed, the interactome of ERβ5 has been described in glioblastoma cells, and it has been revealed that this receptor can interact with mechanistic target of rapamycin kinase (mTOR) or eIF-2-alpha kinase activator GCN1 to promote cell migration and invasion [45]. We did not observe specific effects of ERβ5 or ERβ2 on HGrC1 cell growth, but we cannot exclude that these receptors have a role in GC migration during tumorigenesis. However, testing this hypothesis requires the development of a novel tumoral cell line with functional active ERs.

Excessive GC apoptosis might disturb folliculogenesis and lead to pathophysiology. Follicular development disorder is the commonest endocrinopathy in women, which leads to anovulatory infertility, also known as polycystic ovary syndrome (PCOS). The cause of PCOS remains largely unknown, but extensive evidence suggests an intrinsic ovarian abnormality [46]. Studies on PCOS patients indicated a significant imbalance between apoptotic and proliferation rates in GCs of these patients [47]. Interestingly, in addition to aberrant expression of gonadotropin receptors, AR, steroidogenic enzymes, and AMH receptors, altered ER expression was reported [48,49,50,51]. Hence, additional studies aimed at determining the mRNA levels of the pro-apoptotic ERβ1 and ERβ4 isoforms in PCOS patients would be important to provide new information about the influence of ERβ isoforms on the follicular dysregulation observed in this pathology.

Our study highlights the possible interdependent mRNA abundance of ERβ isoforms in hGCs and the role of E2 in upregulating ERβ4 and ERβ5 mRNA levels. We also demonstrated the pro-apoptotic activities of the ligand-sensitive and -insensitive ERβ1 and ERβ4 isoforms in HGrC1 cells, respectively. E2 would therefore be able to modulate GC fate by specifically regulating the relative transcript abundance of ERβ isoforms to modulate the balance between pro-apoptotic and non-apoptotic ERβ molecules. A better understanding of the molecular pathways that control ERβ isoform transcript levels and their interplay may have therapeutic potential in the treatment of patients with abnormal follicular development.

## 4. Materials and Methods

### 4.1. Patient Population

In total, 49 patients (median age = 34.2 ± 4.7-year-olds; median AMH levels = 2.7 ± 1.5 pmol/L and antral follicular count (AFC) = 19.9 ± 8.5) undergoing in vitro fertilization (IVF) at Antoine Béclère Hospital (Clamart, France) were included in this study. All women met the following inclusion criteria: (1) between 20 and 40 years; (2) both ovaries present, with no morphological abnormalities, adequately visualized in transvaginal ultrasound scans; (3) menstrual cycle length ranging between 26 and 30 days; (4) no current or past diseases affecting ovaries or gonadotropin and sex steroid secretion, clearance, or excretion; (5) no clinical signs of hyperandrogenism; and (6) no polycystic ovary morphology at ultrasonography. Infertility was due either to tubal or sperm abnormalities. The investigation received the approval of our internal institutional review board, IRB Blefco-IORG0010582, and is registered under number “2021-1”. All women signed an informed consent before participating.

### 4.2. Human Granulosa-Lutein Cells Isolation, Culture, and Treatment

Human granulosa-lutein cells (hGCs) were collected from preovulatory follicles during oocyte retrieval for IVF. After oocyte isolation, follicular fluids (FFs) from follicles of each patient were pooled. hGCs were then purified as previously described [46]. Briefly, FF was centrifuged through a one-step density Percoll gradient (*vol/vol*, Dulbecco’s phosphate-buffered saline [DPBS]/Percoll (Gibco, Thermo Fisher Scientific, Les Ulis, France)) at 4000× *g* for 15 min to remove red blood cells. hGCs were collected at the interface, washed with DPBS, resuspended with Dulbecco’s modified Eagle medium (DMEM)/nutrient mixture F-12 Ham (DMEM/F12) (1:1) (Gibco, Thermo Fisher Scientific) supplemented with 10% fetal calf serum (FCS) (Gibco, Thermo Fisher Scientific) and antibiotics (Penicillin-Streptomycin [5000 U/mL] (Gibco, Thermo Fisher Scientific)), and seeded at 1.5 × 10^5^ cells per well in 12-well plates (for mRNA expression analysis) or 2 × 10^4^ cells per well in 96-well plates (for transactivation analysis) in growing media at 37 °C with 5% CO_2_. Twenty-four hours after seeding in 12-well plates, hGCs were cultured in Phenol red-free DMEM/F12 (Gibco, Thermo Fisher Scientific) supplemented with 10% charcoal-dextran (Sigma-Aldrich) stripped FCS and antibiotics for 24 h. Cells were then treated with either 10 nM 17β-Estradiol (Sigma-Aldrich Inc., St. Louis, MO, USA) (stock solutions at 10 mM in ethanol, diluted in the culture medium to the desired final concentration) or the same volume of ethanol (solvent vehicle, V) for an additional 24 h. Depending on the number of isolated hGCs, we performed a single (V or E2) or four treatments (V or E2).

### 4.3. HGrC1 Cells Culture

The HGrC1cell line is an immortalized granulosa cell line established from the ovary of a 35-year-old woman [23]. HGrC1 cells derive from granulosa cells of antral follicles with a diameter of–3–5 mm. They were immortalized using Tet-Off-inducible lentivirus system to introduce human telomerase reverse transcriptase (hTERT) (activate telomerase) as well as mutant cyclin-dependent kinase (CDK4), cyclin D1, and human papillomavirus type 16 (HPV16) oncogenes E6E7 to inactivate both tumor suppressors p53 and retinoblastoma pRB [23]. HGrC1 cells were cultured in DMEM/F12 containing 10% FCS and antibiotics, and maintained at 37 °C with 5% CO_2_. This cell line harbors numerous hallmarks of GC [23].

### 4.4. Construction of FLAG-Tagged Fusion Protein Expression Vectors 

pcDNA4 plasmids with cDNA inserts encoding the ERβ1, ERβ2, ERβ4, or ERβ5 isoform gene, kindly provided by Dr Ricky Y.K. Leung (Department of Environmental Health, Center for Environmental Genetics, University of Cincinnati, Cincinnati, OH, USA.) [15], were used to sub-clone ERβ cDNA into the pFLAG-CMV2 vector (Sigma-Aldrich) by polymerase chain reaction (PCR) using specific primers containing *NotI* and *BamHl* restriction sites to facilitate cloning: Forward: 5′-GGGGGGGCGGCCGCATGGATATAAAAAACTCACC-3′; Reverse ERβ1: 5’-CCCCCCGGATCCTCACTGAGACTGTGGG-3’; Reverse ERβ2: 5’-CCCCCCGGATCCTCACTGCTCCATCG-3’; Reverse ERβ4: 5’-CCCCCCGGATCCCTAAGATAACTTCAAA-3’; Reverse ERβ5: 5’-CCCCCCGGATCCTTAGGGCGCGTACC-3’. We mutated the ERβ cDNA starting site (underlined) and added a base (a G residue on the direct strand, in italic) just before this site to get Flag tag fused in frame with ERβ isoform genes, using the QuickChange mutagenesis kit (Stratagene, Fisher Scientific) with pairs of complementary mutagenic primers: Forward: 5′-CTT GCG GCC GCG GTG GAT ATA AAA AAC-3′; Reverse: 5′-GTT TTT TAT ATC CAC CGC GGC CGC AAG-3. The fusion protein open-reading frame as well as the whole sequence were checked by DNA sequencing (Eurofins Genomics, Les Ulis, France). Large preparations of plasmid DNA were carried out using the Nucleobond AX2000 kit (Macherey-Nagel, Hoerdt, France), and their concentrations were assessed at 260 nm using a Nanodrop (Thermo Scientific Nanodrop 2000).

### 4.5. Transactivation Assays of HGrC1 Cells and Human Primary Granulosa Cells

An empty pGL2-Firefly luciferase vector (pGL2), a pGL2 vector containing three copies of vitellogenin estrogen-response element sequence (ERE-Luc) upstream of luciferase (Addgene, Watertown, MA 02472, USA), and a pRL-SV40-Renilla luciferase (pRL-Renilla) vector (Promega, Charbonnières les bains, France) were used in the reporter assays. HGrC1 cells and hGCs were cultured in Phenol red-free DMEM/F12 supplemented with 10% charcoal-dextran stripped FCS and antibiotics. Cells were then transiently transfected with different vectors (see below) using LipofectamineTM 3000 reagents (Invitrogen, Thermo Fisher Scientific) following the manufacturer’s protocol.

#### 4.5.1. HGrC1 Cells

For one well of a 96-well plate, 50 ng of pGL2 vector or ERE-Luc vector were cotransfected with 27 ng of Flag-ERβ1 together with 27 ng of Flag (equivalent mass as that used in Flag alone (C) condition), Flag-ERβ2, Flag-ERβ4, or Flag-ERβ5 vector (1:1 ratio) using 0.3 µL of LipofectamineTM 3000 and 0.2 µL of P3000 reagents. Promoter reporter plasmids and the empty vector (Flag) for isoform overexpression were equivalent. Each transfection mixture included pRL-Renilla vector (15 ng) as a control for transfection efficiency.

#### 4.5.2. hGCs

For one well, 100 ng of pGL2 vector or ERE-Luc vector were cotransfected with pRL-Renilla vector (16 ng) using 0.3 µL LipofectamineTM 3000 and 0.1 µL of P3000 reagents.

Twenty-four hours later, HGrC1 cells or hGCs were treated for an additional 24 h with either solvent vehicle (V) or 10 nM E2, in the presence of absence of 1 µM ER degrader (SERD) Fulvestrant (Sigma-Aldrich). Standard dual-luciferase assays were performed on the cell lysates, with luciferase assay and Stop & Glo dual-luciferase reporter assay reagents (Promega). Luminescence signals were measured in a Lumat LB 9507 luminometer (Berthold, Germany). The transactivation activity resulting from Firefly was normalized to that of Renilla luciferase and presented in an arbitrary unit as the fold of the relevant control. All transfections were performed in six replicates.

### 4.6. Nucleofection of HGrC1 Cells

For cell viability and apoptosis assays, HGrC1 cells were nucleofected using materials supplied in the Amaxa Cell Line Optimization Nucleofector Kit™ (Lonza, Cologne, Germany). HGrC1 cells were grown to a confluence of 70–80%. Following trypsinization, the cell number was monitored and 1 × 10^6^ cells were suspended in 100 μL of Cell Line Nucleofector Solution V (Amaxa) containing 4 µg of vector (Flag, Flag-ERβ1, Flag-ERβ4, or Flag-ERβ5) in an Amaxa-certified cuvette. Immediately following pulsation, 500 μL of pre-warmed DMEM/F12 supplemented with 10% FCS and antibiotics were added to each cuvette. The nucleofected HGrC1 cells were then transferred into 24-well or 96-well plates containing fresh, pre-warmed DMEM/F12 supplemented with 10% FCS, and antibiotics, and then maintained at 37 °C/5% CO_2_. Assays were performed after 24 h of recovery. This method exhibited ~70% transfection efficiency in HGrC1 cells, when using a GFP reporter gene.

### 4.7. Cell Viability and Apoptosis Assays

#### 4.7.1. Cell Viability MTT Assay

HGrC1 growth was tested by 3-(4,5-dimethylthiazol-2-yl)-2, 5-diphenyltetrazolium (MTT, Sigma-Aldrich) assay. First, 1 × 10^6^ HGrC1cells were electroporated and seeded at a density of 4 × 10^4^ cells per well in 24-well plates in 10% FCS growth medium. At each time studied (24 and 48 h), MTT (5 mg/mL in DPBS) reagent was added in each well at a final concentration of 0.9 mg/mL, and cells were incubated for 2 h at 37 °C/ 5% CO_2_. The supernatant was removed, and the cells were lysed with 200 µL/well DMSO for 10 min. Absorbance at 575 nm (OD_575nm_) was recorded using an ELISA plate reader (Flexstation3, Molecular devices). Fold change in growth was expressed as the ratio of the OD_575nm_ value at 48 h post-transfection to the one measured at 24 h post-transfection. Each experiment was performed four times, and data were reported as means ± SEM from four identical wells.

#### 4.7.2. Early Apoptosis Assay

HGrC1 cell apoptosis was examined with multiplex RealTime-Glo™ Annexin V Apoptosis and CellTiter-Glo^®^ 2.0 Cell Viability Assays (Promega) according to the manufacturer’s protocol. Briefly, 1 × 10^6^ HGrC1 cells were electroporated and seeded at a density of 4.8 × 10^4^ cells per well in 96-well plates in Phenol red-free DMEM/F12 supplemented with 10% FCS and antibiotics. Twenty-four hours after transfection, 2× bioluminescent annexin reagents (Promega) were added to media and the luminescence expressed as a relative light unit (RLU) was read using Flexstation3. Next, cells were lysed by the addition of CellTiter-Glo^®^ 2.0 reagents and 10 min of incubation in the dark before luminescence measurement using Flexstation3. Luminescence associated with the RealTime-Glo™ Annexin V Apoptosis was quenched by the lytic detergents in the CellTiter-Glo^®^ 2.0 Reagent. Therefore, the second luminescent signal measured is directly attributable to the ATP content (viable cells). The ratio of the RLU of Annexin V binding to the one measured with CellTiter-Glo^®^ 2.0 reagents (total cell number) was expressed as arbitrary units. The data represent means ± SEM of four replicates from four independent experiments.

### 4.8. Quantitative Reverse Transcription Polymerase Chain Reaction (RT-qPCR)

Total RNA was extracted using TRIzol reagent according to the manufacturer’s protocol, and 1 µg was used for complementary DNA (cDNA) synthesis by reverse transcription SuperScript II reverse transcriptase (Invitrogen, Thermo Fisher Scientific) using random primers (Promega) according to the manufacturer’s instructions. Quantitative PCR was performed using a standard SYBER premix Taq kit protocol in 384-well plates and the LightCycler 480 Instrument (Roche Diagnostics, Meylan, France). Each 10-µL PCR reaction consisted of 5 µL of SYBER Green PCR Master Mix (Roche) containing 0.1 µM specific primers and 5 µL of cDNA. Primers used are listed in Table 1.

**Table 1:** Primers used for quantitative real-time RT-PCR.

Target Gene (Protein) Accession No.	Forward Primer (*Tm*) Reverse Primer (*Tm*)	Product Size, Amplification Efficiency
*ESR1* (ERα) NM_001122740	5’-CCA CCA ACC AGT GCA CCA TT-3’ (59.4 °C) 5’-GGT CTT TTC GTA TCC CAC CTT TC-3’ (60.6 °C)	116 bp, 97.2%
*ESR2* v1 (ERβ1) NM_001437	5’-GTC AGG CAT GCG AGT AAC AA-3’ (57.3 °C) 5’-GGG AGC CCT CTT TGC TTT TA-3’ (57.3 °C)	192 bp, 90.9%
*ESR2* v2 (ERβ2) NM_001040275	5’-TCT CCT CCC AGC AGC AAT CC-3’ (61.4 °C) 5’-GGT CAC TGC TCC ATC GTT GC-3’ (61.4 °C)	162 bp, 96%
*ESR2* v4 (ERβ4) NM_001214902	5’- GTG ACC GAT GCT TTG GTT TG-3’ (57.3 °C) 5’-ATC TTT CAT TGC CCA CAT GC-3’ (55.3 °C)	210 bp, 96%
*ESR2* v5 (ERβ5) DQ838583.1	5’-GAT GCT TTG GTT TGG GTG AT-3’ (55.3 °C) 5’-CCT CCG TGG AGC ACA TAA TC-3’ (59.4 °C)	177 bp, 95%
*GAPDH* (GAPDH) NM_002046.7	5’-TCC CTG AGC TGA ACG GGA AG-3’ (61.4 °C) 5’-GGA GGA GTG GGT GTC GCT GT-3’ (63.5 °C)	227 bp, 98.5%

The cycling parameters were 95 °C for 10 min, 40 cycles of 95 °C for 10 s, as well as final melting step with slow heating from 65 °C to 95 °C. All reactions were run in triplicate within a single run, and the negative control reactions without reverse transcription reaction and template were also performed. A standard curve consisted of four 1:10 serial dilutions of hGCs cDNA was performed for each set of primers. The primer pair efficiency was obtained from the standard curve experiment where a series of dilution of the same sample was correlated to the Ct values. Quantification of the amount of target genes mRNA was calculated relative to that of the GAPDH (glyceraldehyde-3-phosphate dehydrogenase) normalizer gene and expressed as relative units.

### 4.9. Immunofluorescence of Transfected HGrC1 Cells

HGrC1 cells were seeded into 12-well plates containing 14-mm glass coverslips (Dutcher, Brumath, France). Flag-ERβ1, Flag-ERβ2, Flag-ERβ4, or Flag-ERβ5 were overexpressed in HGrC1 cells using Lipofectamine™ 3000 reagents, according to the manufacturer’s instructions. Briefly, for each 14-mm glass coverslip, 3.54 µL of Lipofectamine™ 3000 were diluted in 59 µL of OptiMEM (Gibco, Thermo Fisher Scientific) and, after 5 min of incubation, added to 59 µL of OptiMEM containing 1.4 µg of DNA and 2.36 µL of P3000 reagent. The DNA-lipid complex was allowed to form for 15 min at room temperature, and the 118-µL mixture was then added to the culture. Twenty-four hours after transfection, cells were fixed with 4% (*wt/vol*) paraformaldehyde for 15 min at room temperature. HGrC1 cells were then permeabilized with 0.1% Triton X-100 (Sigma-Aldrich) in DPBS for 5 min and blocked for 1 h with 3% (*w/v*) bovine serum albumin (Sigma-Aldrich) in DPBS. The coverslips were then incubated for 1 h at room temperature with monoclonal anti-Flag M2 primary antibody (Sigma-Aldrich) diluted 1:2000 in blocking buffer. After five washes with 0.1% Tween 20 in DPBS, the coverslips were incubated for 1 h with an Alexa Fluor^®^ 488-conjugated anti-mouse IgG antibody (Molecular Probes, Thermo Scientific, Illkirch, France) diluted 1:1000 in blocking buffer. The coverslips were finally washed five times with 0.1% Tween 20 in DPBS and cell nuclei were counterstained with 300 nM 4’,6-diamidino-2-phenylindole (DAPI) (Sigma-Aldrich) and preserved in Prolong antifade Gold reagent (Life Technology, Thermo Fisher Scientific). Fluorescent signals were captured and evaluated with a fluorescence microscope (Nikon Eclipse 90i) equipped with an AxioCam HRc (Zeiss, Marly le Roi, France). Different fields were photographed at ×20 magnification.

### 4.10. Statistical Analysis

Results are presented as the mean ± standard error of the mean (SEM) of at least three independent experiments. Statistical analyses were performed using GraphPad Prism 5.0 software (GraphPad Software, La Jolla, CA, USA). We used non-parametric statistic either because our data did not follow a normal distribution, or our sample size was small. Wilcoxon matched-pairs tests and Mann–Whitney U tests were used to compare cells treated with either vehicle (V) or E2, from related or independent samples, respectively. Continuous variables were compared by Spearman’s rank order test. *p* ≤ 0.05 was considered statistically significant.

## Data Availability

Statement original data are with the authors.
